# Erosion of Amazonian mangroves over peatlands leads to soil carbon loss

**DOI:** 10.7717/peerj.21044

**Published:** 2026-04-01

**Authors:** Angelo F. Bernardino, Vitor Leonardo Amaral Rodrigues, André Vassoler, Gabriel C. Coppo, Carla Frecchiami de Oliveira Pacheco, Daniela Y. Gaurisas, Marcelo Cancela Lisboa Cohen, Fellipe Alcantara de Oliveira Mello, Mauricio Roberto Cherubin, Tiago O. Ferreira

**Affiliations:** 1 Departamento de Oceanografia, CCHN, Universidade Federal do Espírito Santo, Vitoria, Espirito Santo, Brazil; 2 CCARBON-Center for Carbon Research in Tropical Agriculture, Universidade de São Paulo, Piracicaba, São Paulo, Brazil; 3 Universidade Federal do Pará, Belém, Para, Brazil; 4 Escola Superior de Agricultura Luiz de Queiroz, Universidade de São Paulo, Piracicaba, São Paulo, Brazil; 5 Research Center for Greenhouse Gas Innovation (RCGI), Universidade de São Paulo, Piracicaba, São Paulo, Brazil

**Keywords:** Wetlands, Blue carbon, Mangroves, Atlantic, Soil carbon, Erosion

## Abstract

**Background:**

Mangrove forests are coastal ecosystems with high soil carbon stocks as a result of intense forest productivity and saturated soils that favor organic preservation for long periods of time. In some regions, the overlap of mangroves with peatlands revealed large soil carbon stocks with great conservation interest. Coastal erosion significantly threatens mangrove forests globally, yet carbon emissions from such losses, especially over peatlands, remain underexplored. Although peatlands are abundant in South America, their overlap with mangroves along the Brazilian Amazon coast has never been fully explored.

**Methods:**

We explored mangroves in the Amazon coast of Brazil that were predicted to occur over coastal peatlands, and estimated their soil carbon stocks. We then overlapped the peatland and mangrove distribution on the Amazon coast to quantify their extent, estimated the potential emission factors associated with erosion and loss of their soil carbon stocks.

**Results:**

We discovered shallow organic peat layers beneath Brazilian Amazon mangroves and estimated that a significant portion of Brazil’s mangroves overlap with peat deposits. Here we conducted soil sampling and carbon stock analyses on Maracá Island, revealing peat layers 51–71 cm thick with high carbon content. Carbon stock losses from eroded mangrove peatlands were estimated at 100.5 ± 33.6 Mg C ha^−1^, representing 72% of soil carbon stocks. These findings highlight the substantial carbon reservoir in Amazonian mangrove peatlands and their vulnerability to erosion-driven carbon release. Recognizing mangroves over peatlands as distinct ecosystems is crucial for accurate carbon accounting and informs conservation strategies aimed at mitigating greenhouse gas emissions from coastal erosion in the Amazon.

## Introduction

Mangrove forests are coastal wetlands with rich organic carbon reserves. The carbon stocks in mangroves can vary considerably across coastal geomorphologies, with soil depth, precipitation, tree mass and latitude, and typically accounts for over 50% of the total ecosystem stocks of each site (Rovai et al., 2022; Kauffman et al., 2020). In some regions, mangroves can have disproportionally high carbon stocks, including in areas where these forests overgrow over freshwater peatlands (Sasmito et al., 2020; Murdyiarso et al., 2024; Rodrigues et al., 2026), or in areas where soil geochemical processes that favor their long-term permanence (Ruiz et al., 2024; Pacheco et al., 2026). Although mangrove over peatlands is a common feature (nearly 10% of mangroves) of Indonesian wetlands (Murdyiarso et al., 2024), they have been largely overseen globally although peatlands are known to occupy large areas of major riverine and coastal basins (Gumbricht et al., 2017).

Given the large spatial variability in total ecosystem carbon stocks of mangrove forests, there are challenges in large-scale extrapolations when determining their potential climate benefits and CO_2_ emissions caused by mangrove degradation (Adame et al., 2021). Therefore, the spatial management of blue carbon may depend on detailed (spatially accurate) and field-based mangrove forest emission data where carbon stocks from undisturbed and disturbed forests are compared (IPCC, 2006). Globally, many studies have determined local blue carbon inventories and measured their loss upon mangrove conversion or land use and land cover change (LULCC; Sasmito et al., 2019). The carbon losses from LULCC are typically converted into greenhouse gas (GHG) emissions, also called emission factors, following international protocols, and assuming that the fate of the organic carbon removed from impacted areas cannot be determined (IPCC, 2006). Based on *in situ* data from mangrove-dense areas, a number of countries including Indonesia (Arifanti et al., 2022; Murdyiarso et al., 2024), Australia (Lovelock et al., 2023) and Brazil (Bernardino et al., 2024a), have assembled regional Blue Carbon stocks and LULCC assessments which support the inclusion of mangroves into national policies for mitigation and restoration. In Brazil, the GHG emission rates currently available for LULCC range from 1,228 to 1,390 Mg CO_2_e ha^−1^ (Kauffman et al., 2018; Bernardino et al., 2024a). These emission factors are close to global values for mangroves (Sasmito et al., 2019), and includes mostly forests impacted by shrimp farms, agricultural activities and pastures.

Mangroves are also threatened by natural disturbances, including coastal erosion, but emissions associated with these losses are yet poorly quantified globally (Goldberg et al., 2020). In the Amazon coast of Brazil, which holds nearly 700,000 ha of mangrove forests, coastal erosion has been identified as the main driver of natural mangrove loss in recent decades (Bernardino, Nobrega & Ferreira, 2021; Bernardino et al., 2022; Goldberg et al., 2020). Erosion of mangrove soils may be driven by increased hydrodynamic energy, which alters the balance between sediment deposition and removal. This shift in the depositional–erosional equilibrium, in combination with sea level rise, can accelerate mangrove loss potentially reducing habitat stability and compromising the long-term resilience of these ecosystems (Cohen et al., 2009, 2018). However, as mangrove carbon stocks vary spatially, there is no *in situ* data available to accurately determine the resulting GHG emission factors from erosion effects. Even at a global scale, although the extent of mangrove forest loss can be determined remotely and carbon losses can be estimated based on a gain and loss methods (Lagomasino et al., 2019), the accuracy of carbon being buried in new forests compared to what is lost in eroded areas is limited by the absence of field data. In this study, we aimed to identify carbon stocks of mangroves where they co-occur with shallow peatlands soils on the Amazon coast in Brazil, and estimate their potential distribution. In addition, we estimated the potential emission factors associated with erosion in that region based on the IPCC area loss method.

## Materials and Methods

**Figure 1:**
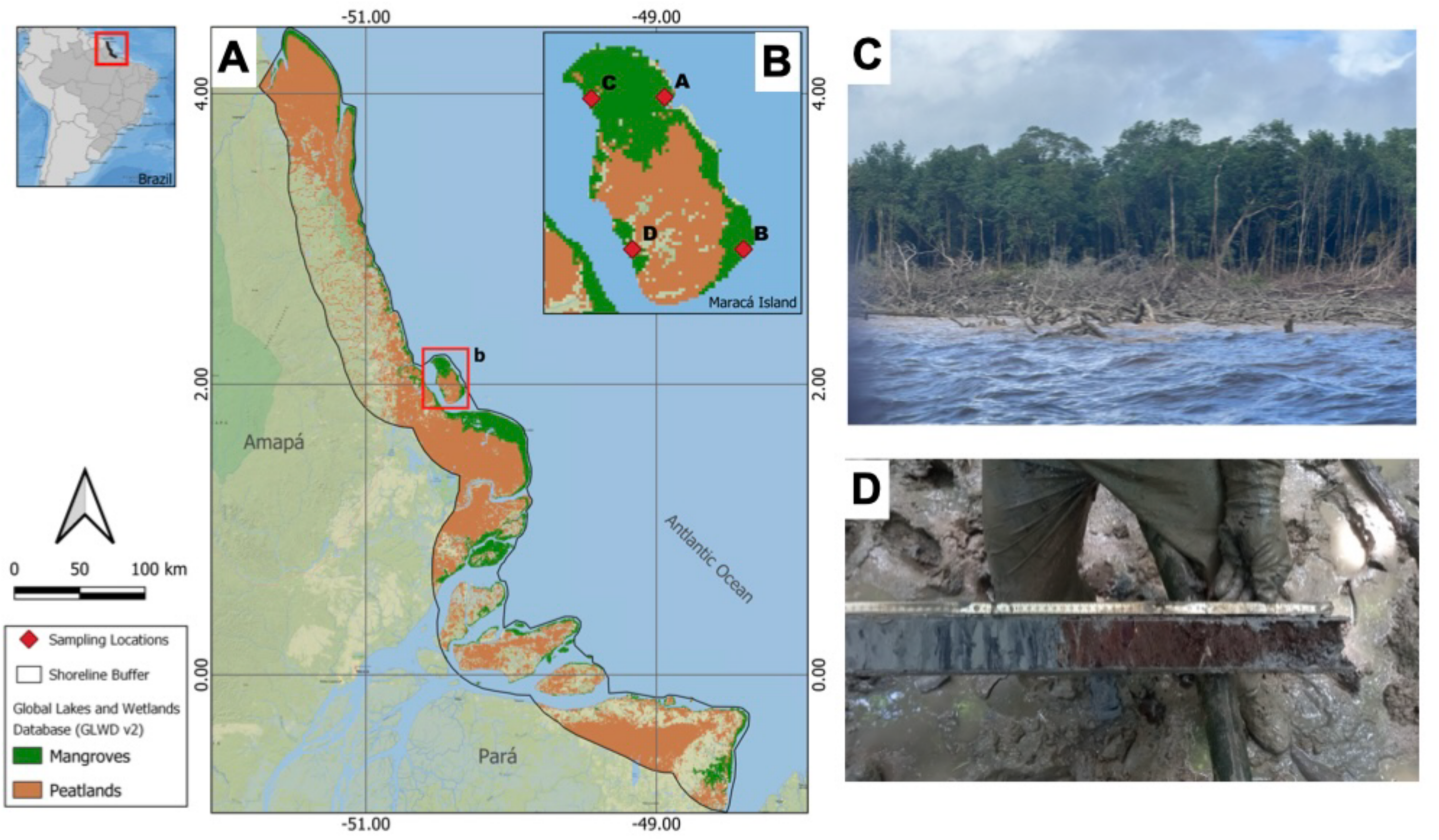
Mapped distribution of mangroves over peatlands in the Amazon coast. Distribution of mangroves (green) and peatlands (light brown) on the Northern Amazon coast, Brazil. (B) Detail showing the sampling sites at Maracá Island in the Brazilian Amazon coast. (C) Eroded areas of Maracá-Jipioca sampled in this study. (D) Detail of peat layer in a soil core sampled in Maracá-Jipioca mangroves. Map component sources: Esri, NASA, NOAA, USGS, Garmin, Foursquare.

**Figure 2:**
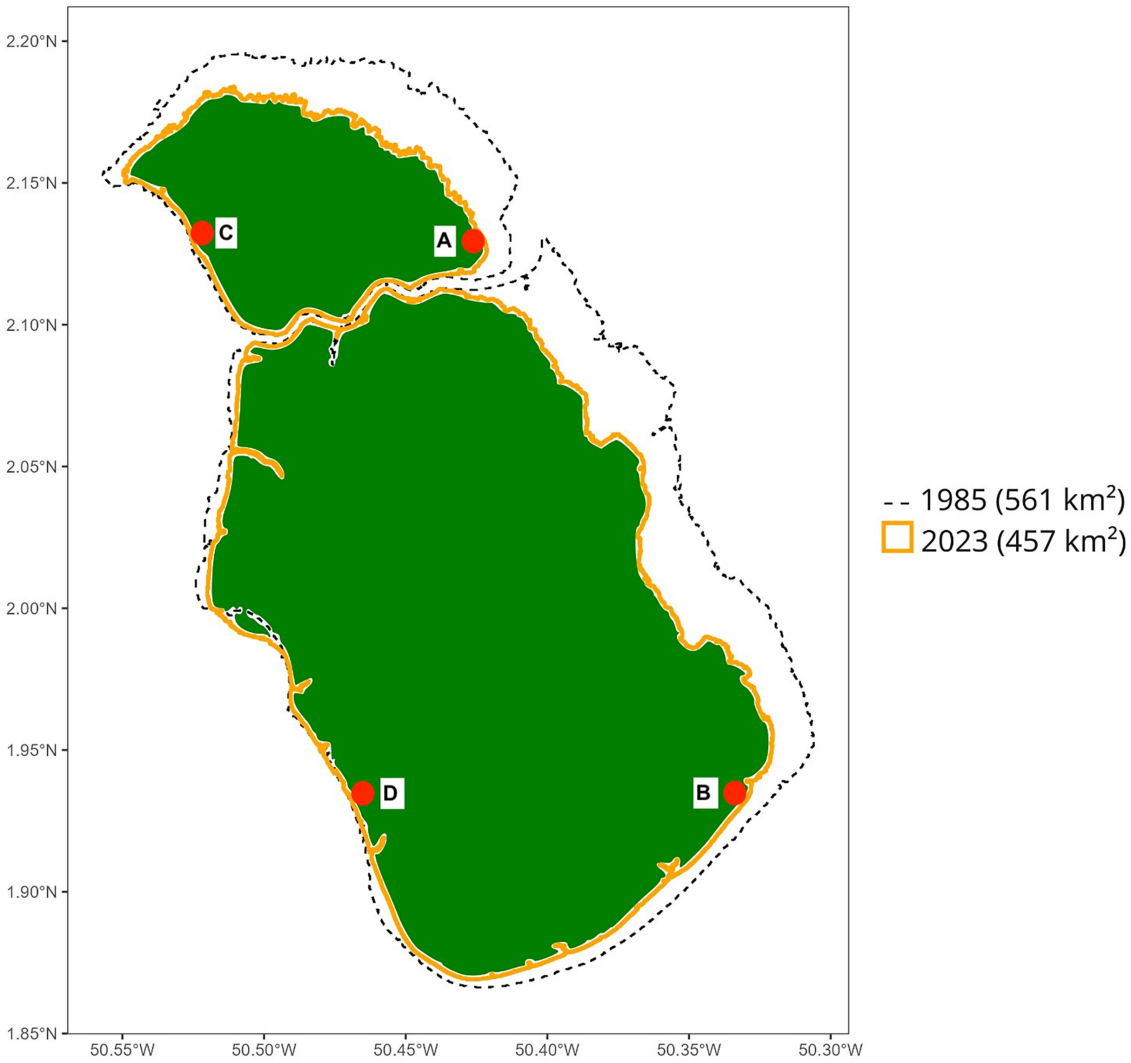
Erosion area on the coast of Maracá Island between 1985 and 2023. Map of Maracá Island indicating the difference on the coastline between 1985 and 2023. The location of mangrove forests sampled (A, B, C and D) indicated by red markers. Map component sources: Esri, NASA, NOAA, USGS, Garmin, Foursquare.

In April 2024, we carried out a 15-day expedition to Maracá Island, located on the Northern coast of the Brazilian Amazon (Fig. 1). Field sampling permit was granted by ICMBio (licence #90274-1). The island is approximately 20 km distant from the Amazon coast and lies within a major South American peatland province (Gumbricht et al., 2017). Coastal erosion is a significant disturbance source of mangroves on the eastern side of the island, whereas the western side is protected and exhibits areas of mangrove progradation in recent years (Fig. 2). As a result, sites A and B represented mangrove forests under influence of erosion, and sites C and D represented controls, in areas with no evidence of erosion process during the last decades according to satellite imagery. Our sampling protocol followed global standard methods used to assess mangrove ecosystem carbon stocks (Kauffman et al., 2018), but here we only report on soil carbon pools down to 200 cm. At each mangrove site, six sub-plots were established 20 m apart along a 100 m transect positioned in a perpendicular direction from the mangrove/estuary ecotone, and soils were sampled to 200 cm depth at each plot. Soil sub-samples were sectioned at 0–15 cm, 15–30 cm, 30–50 cm, 50–100 cm and >100 cm with a 6 cm diameter soil auger. During sampling, visual characteristics of soils (mineral, peat, and mixed) were registered. We determined the maximum soil depth in all plots using a graduated aluminum probe. The soil carbon pools were calculated to 2 m when maximum soil depth was over this limit. In the laboratory, samples were dried at 45 °C and weighed to obtain soil bulk density. Subsamples (0.5 mg) were acidified (HCl 1%) in tin or silver capsules to remove inorganic carbon and combusted in an Elementar vario MICRO cube (Elementar Analysensysteme GmbH) coupled to a mass spectrometer VisION IRMS (Sercon Ltd.) at the UC Davis Stable Isotope Facility, USA. Resulting ^15^N/^14^N and ^13^C/^12^C ratios (reproducibility: 70.5% for d^15^N and 70.2% for d^13^C) were obtained from N_2_ and CO_2_ gases. C-isotopic ratios were measured against a Pee Dee Belemnite (PDB) standard for δ^13^C and atmospheric nitrogen for δ^15^N. Results are expressed as delta (δ) notation, where δX (‰) = [(Rsample/Rstandard) − 1] × 103, where R = 15N/14N or R = 13C/12C.

The carbon stocks in each soil strata were determined as a product of soil bulk density and organic carbon concentration, and results were expressed in megagrams of carbon per hectare (Mg C ha^−1^) for the 2-m soil depth layer. Soil salinity (range 0 to 100) and pH were measured from a handheld refractometer and pH meter (ATC–QC-PH-02), respectively. We strived to measure the borehole salinity and to avoid mixing with surface water that was usually lower in salinity due to rainfall. Greenhouse gas emissions from erosion were estimated based on the assumption of loss of the entire soil carbon stocks to a default depth of 2 m of impacted mangroves according to IPCC guidelines (Tier 2). As we cannot compare total ecosystem carbon stocks differences between eroded and control areas directly due to the presence of peat layers (Murdyiarso et al., 2024), we used the peat layer as a temporal marker and estimated the differences in carbon stocks of the mangrove mineral layer above the peat. The ecosystem losses are reported as potential CO_2_ emissions, or CO_2_ equivalents (CO_2_e) obtained by multiplying C values by 3.67, the molecular ratio of CO_2_ to C. While reported as the CO_2_e, these estimates account only for changes in ecosystem C *in situ*.

The peatland and mangrove areas on the coast of Amapá and Pará States were quantified using the Global Lakes and Wetlands Dataset–GLWD (Lehner et al., 2025). The GLWD is a representation of inland surface water extents and their classification, generated by the harmonization of the latest ground and satellite-based data products. The dataset classified two types of peatlands (forested and non-forested), which were combined in this study to summarize the total peatland areas. Following the predicted occurrence and extent of mangroves on the Amazon coast (Bunting et al., 2022), we used a 50 km buffer starting from the coastline to the continent to mask the GLWD raster dataset and calculate the areas of each vegetation class (mangroves and peatlands). The co-occurring areas of mangroves next to peatlands were then assumed to represent the mangrove over peatland area. The physicochemical differences among the three soil strata (above peat, peat, and below peat) was assessed through a principal component analysis (PCA) using standardized data. The analysis was conducted in R version 4.2 (R Core Team, 2024).

## Results

The sampled mangrove forests along the coastal island of Maracá in Northern Brazil represented areas under long-term coastal erosion (sites A and B; Fig. 1) and areas that have experienced forest accretion during the last decades (Sites C and D; Figs. 1, 2). All mangrove sites were dominated by typical mangrove species, including *Rhizophora mangle* and *Avicennia* sp., in a similar way to other sites sampled in this region (Kauffman et al., 2018; Bernardino et al., 2024b). The studied area was never sampled in detail but is of great interest as these mangroves fall within a massive peat accumulation in South America (Gumbricht et al., 2017). Our exploration of eroded sites revealed layers with organic soils (*i.e*., peat) with a soil carbon content range of 15% to 46% at soil depths of 30–100 cm (Fig. 1). In addition, these peat layers were not found in the two sampling sites (C and D; Fig. 1) at the western side of the Maracá Island (sampled to a depth of 200 cm), suggesting that ongoing coastal erosion is removing surface mineral mangrove soils and gradually exposing the buried peat layers. The two eroded sites (sites A and B; Fig. 1) had peat layers 51 to 71 cm thick and were detected at soil depths of 37 to 130 cm (Fig. 1; Table 1).

**Table 1:** Site metadata and soil organic contents of mangrove over peatlands in the Amazon coast. Sites sampled in Maracá Island, with their soil type based on visual sampling observations. Values in average (±1 standard dev.).

Site (Lat/Long)	Soil salinity (PSU)	Soil pH	Soil type	Layer depth (cm)	Thickness (cm)	Carbon content % (mean)	Bulk density (g cm^−3^)	delta ^13^C	delta ^15^N	C/N ratio	Carbon mass (Mg/ha)
A (02°08.3586/ −50°25.7111)	24	6.9	Mineral	0–37.7	38	1.2	0.79	−26.4 (2.3)	2.5 (0.6)	11.4 (2.7)	46.3
			Peat	37.8–88.5	51	31.9	0.16	−28.2 (0.7)	0.1 (0.4)	22.2 (2.0)	255.5
			Mineral/mixed	88.6–200	111	2	1.26	−29.2 (0.5)	1.2 (0.8)	17.1 (4)	248.6
B (01°56.3018/ −50°19.7541)	11	6.8	Mineral	0–129	129	1.6	0.93	−27.6 (0.7)	3.0 (0.8)	10.7 (3)	151.6
			Peat	130–200	71	32	0.20	−27.7 (0.5)	0.4 (0.2)	21.6 (3.4)	501.1
C (02°07.7854/ −50°31.4362)	5	6.6	Mineral	0–200	200	1.0	1.16	−26.6 (1.1)	4.0 (0.6)	7.5 (2.2)	135.8
D (01°56.6498/ −50°28.4811)	11	6.9	Mineral	0–200	200	0.9	0.95	−26.3 (1.2)	3.8 (0.4)	9.2 (1.5)	161.4

**Figure 3:**
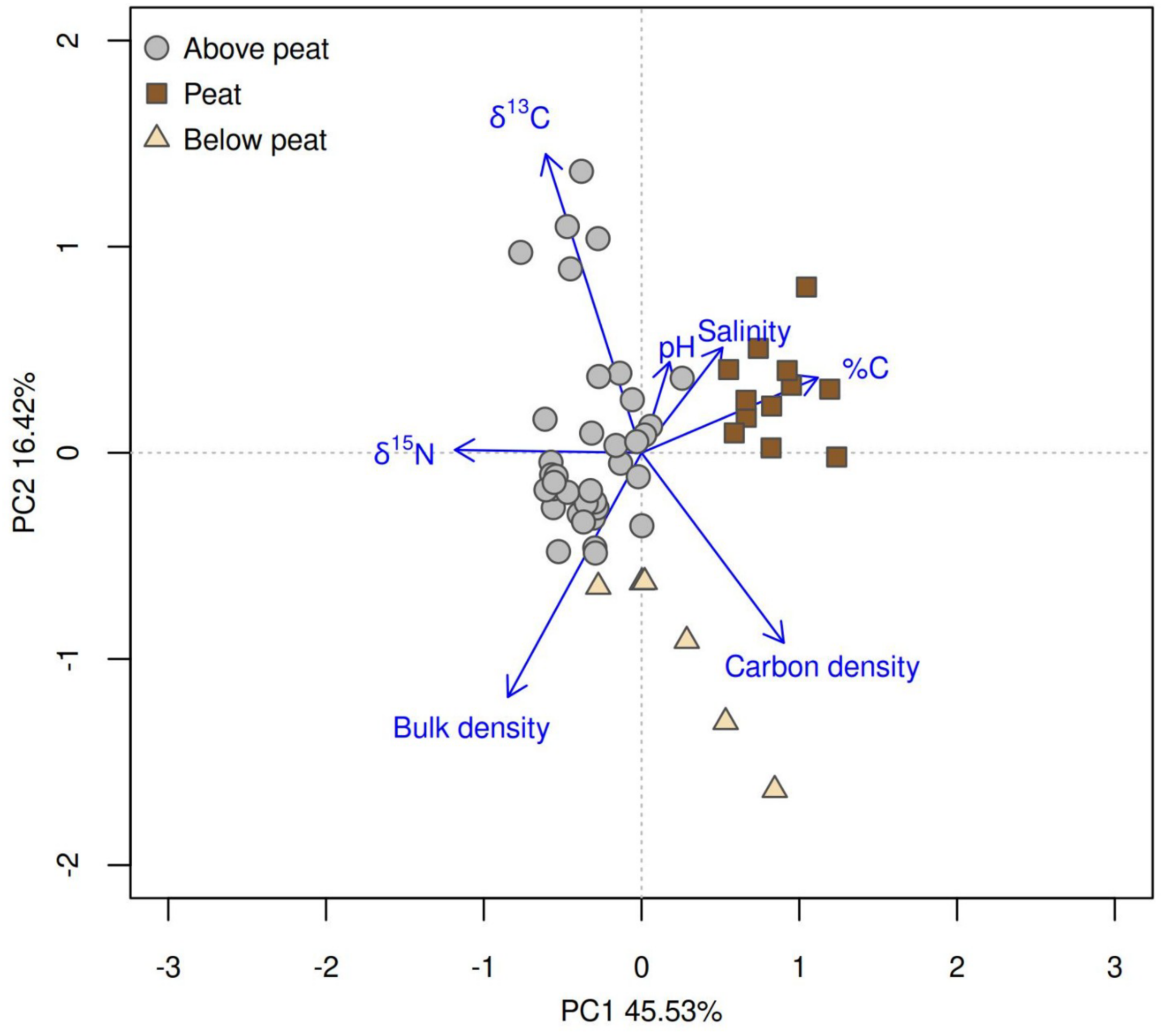
Isotopic signatures of organic carbon and nitrogen from mangroves over peatlands. Principal component analysis of soils sampled in mangroves of Maraca Island, indicating separation between peat and mineral layers based on their soil %C, δ¹³C, δ^15^N, carbon density, and bulk density.

The SOC contents in the mangrove peat layers had an average carbon content (32% C_org_; Table 1) that is over 25 times the content of mangrove soils sampled above or below these peat layers (1–1.2% C_org_). The bulk density of the peat layers (0.16–0.2 g cm^−3^) resembles peat accumulations in the lower Amazon basin and globally (Kauffman et al., 2024; Dargie et al., 2017). We observed that mineral soils above and below peat layers had isotopic signatures indicating a mix of marine and autochthonous sources (*i.e*., higher δ^13^C and δ^15^N), which contrasted with peat (−28.2 < δ^13^C‰ < −27.6; 0.1 < δ^15^N‰ < 0.4; and C/N > 20; Table 1; Fig. 3) with signatures indicating herbaceous C3 plants (Cohen et al., 2015).

To better understand and determine their potential distribution in the Amazon, we overlaid a peatland cover map onto the mangrove forest area from the Global Lakes and Wetlands Dataset–GLWD (Lehner et al., 2025)_,_ using a 50 km coastal buffer. By comparing the peatland extent with the mangrove distribution in the Brazilian Amazon coast, we estimate that mangroves on peatlands potentially cover 3,667 km^2^ (367,700 ha), or 46% of the total mangrove area in the northern Amazon coast (Fig. 1).

As one particular effect of coastal erosion is related to the removal of soil layers with distinct bulk densities and carbon contents, we can expect emission rates for mineral soils associated with mangroves to be markedly distinct from peat. The soil carbon stocks of mangroves over peatlands (*N* = 12 plots; 2 m soil depth) ranged from 550 to 652 Mg C ha^−1^, which were over 4-fold higher than mangroves to the west of the island (136–161 Mg C ha^−1^; Table 1). Peat layers comprise over 50% of the soil carbon stocks in areas where they were sampled. Based on the carbon contents and loss of mineral soil layers above the peat (110.1 ± 34.2 cm; Table 2). The soil carbon stocks of the upper 1 m in mangroves were on average 88.2 ± 8.2 Mg C ha^−1^. By converting the carbon stock mass to the estimated depth of mineral soil layers in the eroded area, we estimate that the current emission rates of mangroves influenced by coastal erosion in the region are near 369 Mg CO_2_-eq ha^−1^ (Table 2). This is equivalent to an emission factor of 0.72 for erosion effects on total ecosystem carbon stocks in that region.

**Table 2:** Carbon emission factors for mangroves over peatlands impacted by erosion. Values estimated based on potential soil carbon losses due to erosion of the upper mineral soil layers. Values in Mg C per ha.

	Mean	95% CI	Propagated uncertainty
Minimum peat depth (cm)	89.9	34.2	
Mineral soil layer above peat (cm)	110.1	34.2	
Soil C stocks 1 m (Mg ha^−1^)	88.2	8.2	
Proportional soil C loss (Mg ha^−1^)	100.5	33.6	
Soil C stock 2 m (Mg ha^−1^)	148.6	19.2	
GHG emissions (Mg CO_2_eq ha^−1^)	368.8		49.6
Emission factors from erosion	0.72	0.3	

## Discussion

Peat layers have never been described as a prominent soil feature of coastal mangroves at the Amazon coast, and although paleoenvironmental peat has been documented in the region (Guimaraes et al., 2012), our study is the first to identify peatland accumulations in mangroves under coastal tidal influence and impacted by erosion. The overlap of mangroves with shallow (<100 cm) peat layers in South America partially supports their predicted distribution along wetlands on the Amazon basin (Gumbricht et al., 2017). Peat layers are an important feature of wetlands and significantly contribute to global carbon sequestration (Treat et al., 2019), but the occurrence of peat deposits on the Amazon coast was only identified in two previous studies. Guimaraes et al. (2012) detected the occurrence of peat layers associated with fluvio-lacustrine deposits from the Holocene (~2,700 cal yr B.P.) at soil depths >120 cm under mangrove mineral soils. Peat deposits were also recently reported on tidal freshwater wetlands in the lower Amazon basin that are within 25 km distance from mangrove forests (Kauffman et al., 2024). Our data supports previous paleoenvironmental studies that have suggested a herbaceous origin of the peat in the region from mid-late Holocene paleo deposits (Guimaraes et al., 2012), that markedly differ from mangrove and freshwater sources that were predominant in mineral soils.

The sampled Amazonian mangrove soils to date are predominantly of mineral origin with very low organic carbon contents, likely due to the intense mineral deposition from the Amazon estuary and the limited iron contents, a key element in the stabilization of soil organic carbon (Passos et al., 2023; Bernardino et al., 2024a; Ruiz et al., 2024). The peat from Amazon mangroves differs from mangrove-derived root accumulations observed in the USA (Osland et al., 2020), but there are potential large similarities with Indonesian peatlands under mangroves (Murdyiarso et al., 2024). Isotopic signatures of mangrove peat layers were similar to those in peat deposits sampled on freshwater wetlands in the lower Amazon basin (Kauffman et al., 2024).

There is significant value in estimating the extent of mangrove over peatlands in South America and the Amazon, since this region is a global hotspot of peatland by both area and volume (Gumbricht et al., 2017). Our results suggest that overall, mangrove over peatlands potentially represent nearly 36% of all mangroves in Brazil, highlighting their potential role in large-scale carbon sequestration and the value in mitigation of their loss (Bernardino et al., 2024b). Indonesian mangroves over peatlands were estimated to represent near 10% of the mangrove area (ca. 311,000 ha) and hold elevated carbon stocks (Murdyiarso et al., 2024) with potential implications for their national carbon accounting. Our results offer a first step in recognizing the value of mangroves of peatlands on the northern Amazon coast, and we recognize the uncertainty with the extent of mangrove over peatlands estimates and peat stratigraphy that could only be resolved with field assessments.

Our work suggests that peatlands may be particularly vulnerable to coastal erosion, potentially resulting in high greenhouse gas (GHG) emission rates from ancient peat degradation within the coming decades. The extensive distribution of these peatlands associated with mangroves has major implications for the carbon balance of the Amazon coast (Bernardino et al., 2024b; Rodrigues et al., 2026); and brings an enormous challenge in modelling global emission rates from mangroves on peatlands with heterogeneous carbon contents. We attempted to narrow the large uncertainty on carbon losses and emissions from coastal erosion effects by comparing the soil stock difference between standing forests under erosion with control areas to the west of the island.

The soil carbon stocks of mangroves over peatlands from this study are in the lower range of global tidal peatland forest values (median of 1,979 Mg C ha^−1^, Kauffman et al., 2025). However, these rates would increase by nearly 10-fold with the loss and removal of peat layers, which would increase emissions to 3,365 ± 1,621 Mg CO_2_-eq ha^−1^ with the complete erosion of these peat-mangrove forests. To put the erosion-emission rates into perspective with common LULCC emissions in mangrove land worldwide, those associated with pastures and shrimp farms on the Amazon coast are 1,228 Mg CO_2_-eq ha^−1^, and buffalo pasture degradation of peatlands in the lower Amazon basin release on average 47 Mg CO_2_-eq ha^−1^ yr^−1^ (Bernardino et al., 2024b; Kauffman et al., 2024). Globally, LULCC effects release near 1,468 Mg CO_2_-eq ha^−1^, due to losses of nearly 54% of soil carbon from mangroves (Sasmito et al., 2019).

One pressing question is whether the carbon losses associated with eroded mangroves along the Amazon coast can be offset by the growth and expansion of forests in other areas (Lagomasino et al., 2019; Visschers, Santos & Franco, 2022). In areas of Southeast Asia and Africa, the growth of new mangrove forests was estimated to offset more than 80% of carbon losses from erosion areas, but estimates were based on models for total ecosystem carbon stocks on mineral-dominated soils (Lagomasino et al., 2019). For mineral-dominated soils, our study supports that mangroves in accretion areas (sites C and D) can sustain a similar carbon density to mineral layers of erosion sites. However, the differences in carbon density and emission factors between mangrove on peatlands and mineral-dominated soils will likely make erosion emissions more pronounced than the carbon stocks that could be replaced by natural regrowth or replanting projects in the region. Therefore, estimating loss and gain from forest cover change would greatly propagate errors in areas where peatlands co-occur with mangroves. However, there is potential to combine remote sensing with peatland coverage, coastal vulnerability maps, and field assessments to refine mangrove carbon losses and gains in these areas.

## Conclusions

Our study presents the first step in recognizing mangroves over peatlands as a distinct feature of mangrove ecosystems in the Amazon coast. Based on the overlay of peatland coverage with current mangrove occurrence in the region, mangroves over peatlands potentially cover up to 3,667 km^2^, but it’s true extent requires further validation. Our results suggest that natural erosion on the Amazon coast has an emission factor of 0.72 for soil carbon stocks, which is equivalent to 100.5 ± 33.6 Mg C ha^−1^. Complete removal of these mangroves by erosion can potentially release a significant amount of carbon from the loss of peatlands, which were previously unaccounted for. By providing a potential distribution and emission factors associated with their natural loss due to erosion, we highlight the need for further study of peatlands under mangroves in the Amazonian province, and fill important gaps in the estimation of erosion effects in Brazil, which has been previously indicated as the main stressor to mangrove forests in the country.

## Supplemental Information

10.7717/peerj.21044/supp-1 Supplemental Information 1 Raw soil C dataset. Soil C from mangrove sites sampled

10.7717/peerj.21044/supp-2 Supplemental Information 2 Images mangrove forests studied in Maracá Island. A. Site A; B. Site B; C. Site C, D. Site D

## Additional Information and Declarations

### Field Study Permissions

The following information was supplied relating to field study approvals (*i.e*., approving body and any reference numbers):

Field sampling was approved by ICMBio (Brazilian Ministry of Environment).

### Data Availability

The following information was supplied regarding data availability:

The soil C concentrations from mangrove raw data is available in the Supplemental File.
