# Supplementary material for: Erosion of Amazonian mangroves over peatlands leads to soil carbon loss
Source: PeerJ. 2026 Apr 1;14:e21044. doi: 10.7717/peerj.21044 (PMC13050214; doi:10.7717/peerj.21044)

Supplementary material for Erosion of Amazonian mangroves over peatlands leads to soil carbon loss

Angelo Bernardino et al.

PeerJ

Figure S1. Images mangrove forests studied in Maracá Island. A. Site A; B. Site B; C. Site C, D. Site D
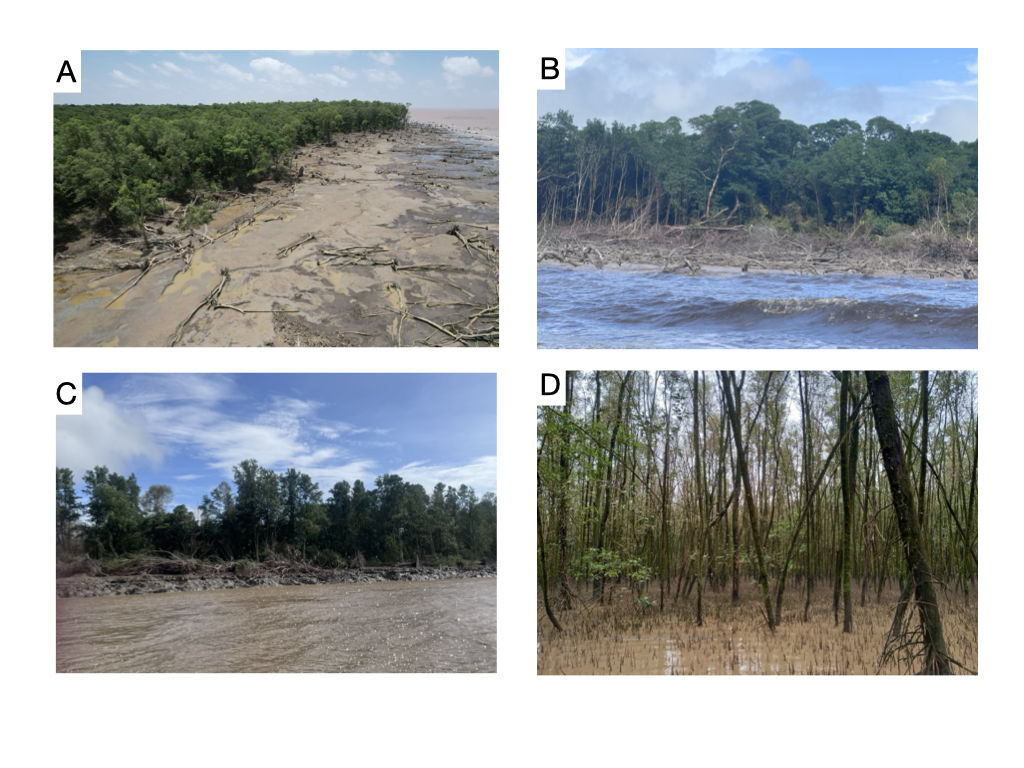

Supplement: Supplemental Information 2 — A. Site A; B. Site B; C. Site C, D. Site D [file peerj-14-21044-s002.docx]
